# Molecular Diagnosis of *Trichomonas vaginalis* in Women and Men: A Systematic Review

**DOI:** 10.1155/jotm/7920568

**Published:** 2026-07-27

**Authors:** Somayeh Mohamadi, Reza Faraji, Saeed Khoshnood, Abbas Maleki, Arezoo Bozorgomid, Saiyad Bastaminejad, Taha Rashidi, Amirhossein Salimi Mansouri, Kosar Khezri, Shahin Seidi, Sadegh Faraji

**Affiliations:** ^1^ Student Research Committee, Ilam University of Medical Sciences, Ilam, Iran, medilam.ac.ir; ^2^ Tuberculosis and Lung Diseases Research Center, Ilam University of Medical Sciences, Ilam, Iran, medilam.ac.ir; ^3^ Clinical Microbiology Research Center, Ilam University of Medical Sciences, Ilam, Iran, medilam.ac.ir; ^4^ Medical Biology Research Center, Health Technology Institute, Kermanshah University of Medical Sciences, Kermanshah, Iran, kums.ac.ir; ^5^ Head of Genetics Department, School of Paramedicine, Ilam University of Medical Sciences, Ilam, Iran, medilam.ac.ir; ^6^ Student Research Committee, Kermanshah University of Medical Sciences, Kermanshah, Iran, kums.ac.ir; ^7^ Research Center for Chronic Inflammatory Diseases, Tehran University of Medical Sciences, Tehran, Iran, tums.ac.ir; ^8^ Zoonotic Diseases Research Center, Ilam University of Medical Sciences, Ilam, Iran, medilam.ac.ir; ^9^ Research Center of Thoracic Oncology (RCTO), National Research Institute of Tuberculosis and Lung Diseases (NRITLD), Shahid Beheshti University of Medical Sciences, Tehran, Iran, sbmu.ac.ir; ^10^ Faculty of Dentistry, Kermanshah University of Medical Sciences, Kermanshah, Iran, kums.ac.ir

**Keywords:** diagnostic accuracy, molecular diagnosis, PCR, sensitivity and specificity, sexually transmitted infection (STI), systematic review, *Trichomonas*

## Abstract

**Background:**

*Trichomonas vaginalis* (*T. vaginalis*) is a human protozoan parasite and the leading cause of nonviral sexually transmitted infections worldwide. Diagnosis of *T. vaginalis* is typically performed using vaginal discharge or urine samples, which are examined via wet prep, culture methods, or molecular tests. Compared to traditional culture‐based methods, molecular diagnostic techniques have shown higher sensitivity, as they allow more precise identification of *T. vaginalis*‐infected individuals. The present review evaluated the molecular diagnosis of *T. vaginalis* in male and female populations.

**Methods:**

A search was performed across international databases, including Google Scholar, Scopus, PubMed, ScienceDirect, Web of Science, Wiley, ClinicalKey, IEEE, EMBASE, Springer, Cambridge, Oxford Journal, Sage, Emerald, and Cochrane. The search included English‐language publications available from inception to August 2025.

**Results:**

In total, 119 studies were eligible for this systematic review. The highest number of studies originated from the United States (*n* = 33), Iran (*n* = 16), and South Korea (*n* = 8). PCR was the most frequently employed molecular technique, reported in 58 studies, followed by real‐time PCR (*n* = 12). 18 studies reported 100% sensitivity and 20 studies reported 100% specificity. Of the included studies, 91 focused only on women, 7 on men, 19 on both genders, and 2 did not specify the participants’ gender. Study design was poorly reported in the majority of studies (*n* = 83), but among those with clear designs, cross‐sectional studies predominated (*n* = 20). Vaginal swabs were the most frequently used sample type.

**Conclusion:**

The sensitivity and specificity of molecular methods for diagnosing *T. vaginalis* vary. However, given their overall superior performance, these techniques should be prioritized for accurate diagnosis and effective treatment of *T. vaginalis* infections.

## 1. Background

*T. vaginalis* is a protozoan parasite that causes a sexually transmitted infection (STI) in humans and impacts the urogenital tract. While it is mostly transmitted through sexual contact, rare cases of nonsexual transmission have been documented. *T. vaginalis* infection is mostly asymptomatic in both men and women, with an estimated 70%–85% of individuals showing minimal or no clinical signs [1, 2]. Symptomatic women may present with vaginal discharge, itching, dysuria, and dyspareunia. Over the past 3 decades, *T. vaginalis* has increasingly been associated with various clinical manifestations, including pelvic inflammatory disease and cervical neoplasia, as well as other reproductive health complications, particularly among women [1]. The incidence of *T. vaginalis* differs by gender and geographic region. *T. vaginalis* is most prevalent among sexually active women, especially those aged 20–45 years and with elevated vaginal pH levels. Vaginal pH > 4.5 is indeed associated with *T. vaginalis* infection, but it is often a consequence of the disruption of normal lactobacillus‐dominant flora rather than an independent risk factor. Globally, *T. vaginalis* infects a majority of individuals (160–180 million) each year, with 154 million cases occurring in resource‐limited regions. In the United States, approximately 8–10 million cases are reported annually, while Europe accounts for nearly 11 million [3, 4]. Notably, the highest incidence rates have been recorded in African countries [5]. Based on the latest annual statistics from the World Health Organization, about 156 million new cases of *T. vaginalis* infection are identified each year among individuals aged 15–49 years, exceeding the incidence of other major STIs such as chlamydia, gonorrhea, and syphilis [1, 6]. A recent study has estimated the overall prevalence of *T. vaginalis* among Iranian women to be around 4.30% [6]. In pregnant women, *T. vaginalis* infection is linked to increased risks of preterm delivery and low birth weight. Among men, the parasite has been implicated in conditions such as urethritis, epididymitis, prostatitis, and impaired sperm motility. Clinical symptoms can help diagnose *T. vaginalis*; however, its definite diagnosis requires the identification of the parasite’s DNA or its trophozoite form [1, 7]. Histological examination, staining procedures, wet mount microscopy, parasite cultivation, immunofluorescence assays, and serological and molecular methods are current diagnostic methods for *T. vaginalis* [8]. Despite their broad use, their sensitivity and specificity are limited. The standard treatment for trichomoniasis is oral metronidazole, but treatment failure may occur due to gastrointestinal side effects, allergic reactions, or drug resistance. In such cases, intravaginal therapies represent a safer alternative by reducing systemic adverse effects. Nonetheless, the existing research on vaginal treatments and preventive strategies for *T. vaginalis* remains inconsistent and lacks a comprehensive evaluation [5]. Accurate diagnosis of *T. vaginalis* is essential for guiding effective treatment decisions. Reliable laboratory diagnostic methods particularly those with high sensitivity and specificity are crucial. In light of a 2025 systematic review and meta‐analysis conducted by Babafemi et al. [9], which focused exclusively on real‐time PCR and excluded other molecular techniques, we undertook this systematic review to evaluate molecular methods available for detecting *T. vaginalis* in both women and men. Therefore, this systematic review aimed to synthesize the available evidence on the performance, application, and geographic/gender distribution of all molecular diagnostic techniques for *T. vaginalis* in women and men.

## 2. Methods

This systematic review was designed in conformity with the protocol and guidelines provided by Preferred Reporting Items for Systematic Reviews and Meta‐Analyses (PRISMA) [10].

### 2.1. Search Strategy

A comprehensive search was conducted to identify all English‐language articles regardless of publication date or geographic origin, from inception to August 2025, that investigated the molecular diagnosis of *T. vaginalis* infection in men and women. In the search, we included multiple databases: Google Scholar, Scopus, PubMed, ScienceDirect, Web of Science, Wiley, ClinicalKey, IEEE, EMBASE, Springer, Cambridge, Oxford Journal, Sage, Emerald, and Cochrane. After assessing the quality of the articles, eligible studies were selected. Additionally, we used MeSH terms and relevant keywords, individually or in combination, with the Boolean operators AND/OR. The keywords included in the search are in Table 1.

**TABLE 1:** Search strategy and terms used to identify studies.

Term	1. Molecular diagnostic techniques, 2. Molecular detection, 3. Molecular testing, 4. Molecular typing, 5. Molecular sensitivity, 6. Molecular identification, 7. Molecular probe, 8. Molecular investigation, 9. Molecular methods, 10. Molecular study, 11. Molecular assays, 12. Molecular characterization, 13. Genome sequence, 14. PCR, 15. Polymerase Chain Reaction, 16. Nested PCR, 17. Multiplex PCR, 18. Multiplex polymerase chain reaction, 19. RT‐PCR, 20. Random amplification polymorphism, 21. PCR‐restriction fragment length polymorphism DNA, 22. RAPD‐PCR, 23. Real‐time PCR, 24. Real‐time Polymerase Chain Reaction, 25. TaqMan, 26. LAMP, 27. Loop‐mediated isothermal amplification, 28. NAAT, 29. Affirm VPIII, 30. Nucleic acid amplification test, 31. Trichomonad detection, 32. *Trichomonas vaginalis,*” 33. Tv.
Strategy	1 or 2 or 3 or 4 or 5 or 6 or 7 or 8 or 9 or 10 or 11 or 12 or 13 or 14 or 15 or 16 or 17 or 18 or 19 or 20 or 21 or 22 or 23 or 24 or 25 or 26 or 27 or 28 or 29 or 30 and 31 and 32 or 33

### 2.2. Study Eligibility Criteria

#### 2.2.1. Inclusion Criteria

Eligible studies were those published in English and comprised original research, cross‐sectional, case‐control, and cohort studies, or short reports. No time restrictions were applied. All selected studies focused on the molecular diagnosis of *T. vaginalis* infection in women and men and met the quality assessment standards.

#### 2.2.2. Exclusion Criteria

The following types of studies were excluded: case reports, review articles, letters to the editor, articles without full text, studies with unclear patient information, conference papers, duplicate publications, non‐English articles (unless accompanied by English abstracts containing the necessary data for this systematic review), animal studies, mathematical modeling studies, and studies employing nonmolecular diagnostic methods for *T. vaginalis*.

### 2.3. Study Selection and Quality Assessment

The study was initiated in accordance with the PRISMA protocol, after defining the research team members (team lead and final reviewer, advisor, reviewer, researcher, data extractor and compiler, and article searcher). Articles retrieved from database searches were collected without time restrictions, and duplicate records were removed using EndNote 21. Two reviewers independently screened the titles and abstracts to identify eligible studies. In cases of disagreement, a third reviewer evaluated the articles. The Joanna Briggs Institute (JBI) Critical Appraisal Checklist [11] was employed to assess the quality of the selected studies and identify potential biases (e.g., selection, performance, detection, attrition, and reporting). The checklist comprised nine items, each with four response options: “yes,” “no,” “unclear,” and “not applicable.” It was specifically designed to evaluate prevalence and descriptive cross‐sectional studies, focusing on elements such as sample size, study population, study setting, and statistical analysis. Based on the total score, studies were classified as good (7–9), moderate (4–6), and poor (0–3) quality. Studies scoring 3 or below were excluded (2 studies). Final assessments were conducted by two independent reviewers, with any discrepancies resolved through consultation with a third reviewer.

### 2.4. Data Extraction

Data were manually extracted and entered into an Excel spreadsheet. Before analysis, the data were verified for accuracy and consistency. Any discrepancies were resolved through consensus among the authors. From each selected study, the following information was collected: article title, first author’s name, year of publication, country of origin, study type, participants’ gender, sample type and quantity, number of positive cases, target gene and sequence, molecular diagnostic method applied for detecting TV, and the reported sensitivity and specificity of the technique. To prevent missing data, the authors of articles whose full texts were unavailable were contacted.

## 3. Results

**FIGURE 1:**
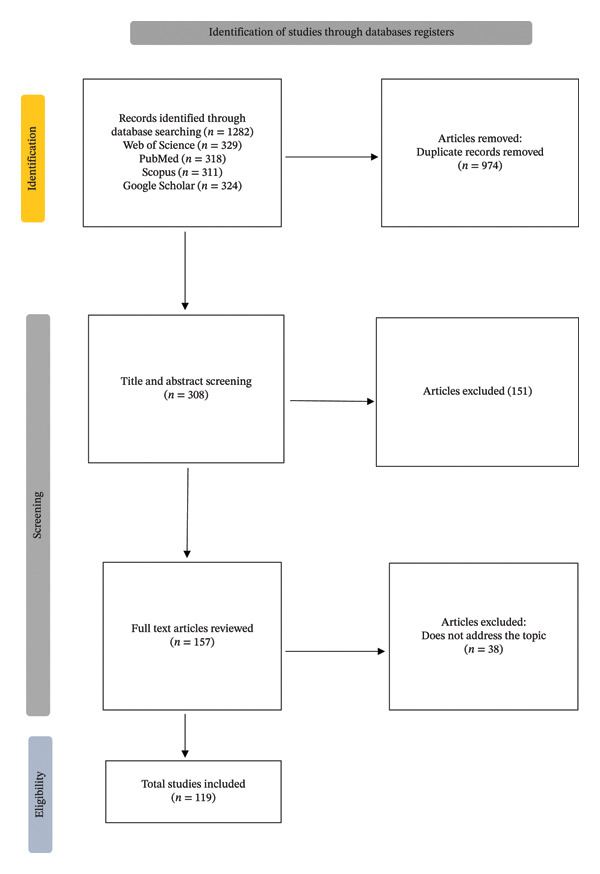
Screening of articles using the systematic review flowchart (PRISMA).

Two independent reviewers extracted the data, achieving a 97% agreement rate for study selection. A total of 1282 articles were identified through database searches. After removing 974 duplicates, 308 studies were screened by title and abstract, resulting in the exclusion of 151 studies due to irrelevance. Full‐text assessment of the remaining 157 articles led to the exclusion of an additional 38 studies that did not meet the eligibility criteria. Ultimately, 119 studies were included in this systematic review (as shown in Figure 1).

Details of the 119 included studies are presented in Table 2. All included studies were published between 1992 and 2025. A heterogeneous mix of study designs was observed. Regarding methodological design, 20 studies were cross‐sectional, 2 were cross‐sectional descriptive, 1 was cross‐sectional observational, 1 was descriptive cross‐sectional, 6 were prospective, 2 were prospective observational, 1 was observational, and 4 were retrospective. Methodological details were unclear in 83 studies.

**TABLE 2:** Characteristics of included studies.

Authors/year/[references]	Country	Type of studies	Sex	Type of specimens (*N*)	Molecular method (target sequence (target gene/primer set))	TV positive (*N*/%)
Adao et al., 2016 [12]	Philippines	NA	F	Vaginal (121)	LAMP and PCR (TvK3 and TvK7)	LAM = 42.06% P and PCR = 7.44%
Alikhani et al., 2021 [13]	Iran	Cross‐sectional	F	Vaginal (1765)	Nested PCR (Tv8s/Tv9R and Tv10s/Tv11R)	21 (1.18%)
Abdolali et al., 2016 [14]	Iran	Cross‐sectional	F	Vaginal (100)	PCR (TVK 3/TVK7)	11 (11%)
Andrea et al., 2011 [15]	USA	NA	F	Vaginal and urine/urethral (766)	Affirm VPIII and ATV (16S rRNA)	5.1%
Bandea et al., 2013 [16]	USA	NA	F	Urine/urethral (406)	PCR and Nested PCR (5.8S rRNA)	PCR = 8 (9.4%) and Nested PCR = 14 (3.4%)
Bokharaei‐Salim et al., 2020 [17]	Iran	NA	F	Vaginal (1550)	PCR (T V K 3/TVK7)	9 (0.6%)
Bui et al., 2023 [18]	USA	NA	F	Vaginal (136)	Real‐time PCR (repeated DNA target)	21
Brown et al., 2004 [19]	USA	NA	F	Vaginal (425)	Affirm VPIII (NA)	30 (7%)
Bahreini et al., 2023 [20]	Iran	Cross‐sectional	F	Liquid cytology/ThinPrep (534)	PCR (T V K 3/TVK7)	26 (4.86%)
Byun et al., 2016 [21]	Korea	NA	F	Vaginal (195)	Affirm VPIII (NA)	1 (0.51%)
Caliendo et al., 2005 [22]	USA	NA	F	Vaginal (524)	Real‐time PCR and PCR	TVK3/TVK4 for real‐time PCR and 18S rDNA for PCR
Cartwright et al., 2013 [23]	USA	NA	F	Vaginal (320)	Affirm VPIII and NAA (rRNA)	54 (Affirm VPIII = 25 and NAA = 53)
Carneiro et al., 2020 [24]	Brazil	NA	F	Cervical/endocervical (NA)	Multiplex PCR (NA)	10.6%
Choe et al., 2013 [25]	Korea	NA	F and M	Urine/urethral, and cervical/endocervical (897)	Real‐time PCR (NA)	8
Conrad et al., 2012 [26]	USA	NA	F	Vaginal (270)	PCR (TVK3/TVK7, V16f‐2/TV16r‐2, and BTub3f/BTUB_Bkmt)	26 (9.6)
Chetty et al., 2020 [27]	South African	NA	F	Vaginal (362)	Real‐time PCR (alpha tubulin 1)	47 (12.9%)
Crucitti et al., 2003 [28]	Ivory Coast	NA	F	Vaginal (416)	PCR (TVA5/TVA6, TVK3/TVK7, BTUB9/BTUB 2, IP1/IP2, and TV1/TV2)	83 (20.0%)
Crucitti et al., 2008 [29]	Congo and Zambia	NA	F	Vaginal (160)	PCR (TVK3⁄TVK 7 and IP1 ⁄ IP2)	151
DeMeo et al., 1996 [30]	USA	NA	F	Vaginal (615)	Affirm VPIII (target RNA)	86 (90.5%)
Dadwal et al., 2023 [31]	India	Prospective	F	Vaginal (1974)	PCR (TVK3⁄TVK 7 and 18S SS‑rRNA)	46
El‐Gayar, et al., 2016 [32]	Egypt	NA	F	Vaginal (110)	PCR (T V K 3/TVK7)	40 (36.3%)
El‐kareem et al., 2024 [33]	Egypt	Cross‐sectional	F	Vaginal (150)	PCR‐RFLP (β‐actin)	13
Fraga et al., 2005 [34]	Cuba	NA	F	Vaginal (40)	PCR (dsRNA genome)	22 (55%)
Fraga et al., 2011 [35]	Cuba	NA	F	Vaginal (37)	RAPD (dsRNA genome)	21 (56.7%)
Field et al., 2018 [36]	UK	NA	F and M	Urine/urethral (4386)	Real‐time PCR (β‐tubulin)	7
Gaydos et al., 2016 [37]	USA	NA	F	Vaginal (990)	APTIMA TV (18S rRNA)	968
Goo et al., 2016 [38]	Korea	NA	F	Vaginal (50)	LAMP, multiplex PCR, and PCR (F3, B3, FIP, BIP, LF, LB)	LAMP = 43, multiplex PCR = 23, and PCR = 40
Hardick et al., 2006 [39]	USA	NA	F and M	Vaginal and urine/urethral (611)	TMA and BTUB PCR (16S rRNA and TVK3/TVK4)	59
Hardick et al., 2003 [40]	USA	NA	F and M	Urine/urethral (253)	BTUB PCR (β‐tubulin)	15
Hathorn et al., 2015 [41]	UK	NA	F and M	Vaginal and urine/urethral (3503)	TMA (18S rRNA)	93
Huh et al., 2019 [42]	Korea	Retrospective	NA	Vaginal and urine/urethral (1106)	Real‐time PCR (SC STD5‐HEX)	154
Huppert et al., 2007 [43]	USA	NA	F	Vaginal and cervical/endocervical (330)	TMA (rRNA)	28 (8.5%)
Heine et al., 1997 [44]	USA	NA	F	Vaginal (61)	PCR (Hin fl)	56 (91.8%)
Herath et al., 2021 [45]	Sri Lanka	Cross‐sectional	F	Vaginal and urine/urethral (385)	PCR (5.8S rRNA and 18S rRNA)	17 (4.4)
Heidary et al., 2013 [46]	Iran	NA	F	Vaginal and urine/urethral (46)	PCR (TVV1)	8 (17.39%)
Ibáñez‐Escribano et al., 2014 [47]	Spain	NA	F	Vaginal (34)	PCR (5.8S rRNA and 18S rRNA)	9 (26%)
González et al., 2024 [48]	Chile	Cross‐sectional descriptive	F	Vaginal and urine/urethral (13)	PCR (β‐globin, ntr4, and ntr6)	9 (69%)
Ingersoll et al., 2008 [49]	Georgia	NA	F	Urine/Urethral (40)	Real‐time PCR (NA)	38
Jones et al., 2013 [50]	South Africa and Brazil	NA	F	Vaginal (South Africa (*n* = 230) and Brazil (*n* = 695))	PCR (NA)	South Africa = 10% and Brazil = 3%
Jordan et al., 2001 [51]	USA	NA	F	Vaginal (552)	PCR (β‐globin)	58 (10.5%)
Knox et al., 2002 [52]	Australia	NA	F	Vaginal, urine/urethral, and cervical/endocervical (318)	PCR (β‐globin)	24.6%
Kaul et al., 2004 [53]	India	NA	F	Vaginal and urine/urethral (1000)	RAPD (OPD 1/OPD 5)	38
Kaydos et al., 2002 [54]	USA	Cross‐sectional	F	Urine/urethral (1513)	PCR (TVK3/TVK7)	NA
Kaydos‐Daniels et al., 2003 [55]	USA	Cross‐sectional	M	Urine/urethral (1225)	PCR (TVK3/TVK7)	NA
Khurana et al., 2019 [56]	India		F	Vaginal (153)	PCR, LAMP, and Xpert TV (18S SS‐rRNA and 18 SSU‐rRNA)	PCR = 31, LAMP = 32, and Xpert TV = 31
López‐Monteon, et al., 2013 [57]	Mexico	Cross‐sectional	F	Urine/urethral (252)	PCR (TV3/TV7)	23.41%
Leli et al., 2016 [58]	Italy	Cross‐sectional, observational	F	Vaginal (1487)	Real‐time PCR (NM)	20 (1.3%)
Liu et al., 2017 [59]	China	NA	F and M	Vaginal and semen/prostate fluid (101)	PCR (18S rRNA)	110
Lee et al., 2012 [60]	Korea	NA	M	Urine/urethral (33)	PCR (E650)	12
Lawing et al., 2000 [61]	USA	NA	F	Vaginal and urine/urethral (190)	PCR (TV3/TV7)	53 (28%)
Lusk et al., 2010 [62]	Australia	Cross‐sectional	F	Vaginal (356)	PCR (TricV‐OF/TricV‐OR and TricV‐IF/TricV‐IR)	17 (4.8%)
Lin et al., 1997 [63]	Taiwan	NA	F	Vaginal (165)	PCR (OPI/OP2 and IP1/IP2)	16
Li et al., 2020 [64]	China	NA	F	Vaginal (NA)	LAMP (AP65)	NA
Muzny et al., 2014 [65]	USA	NA	F & M	Urine/Urethral, and cervical/endocervical (63)	NAAT (NM)	20.2%
Muzny et al., 2016 [66]	USA	Retrospective	F and M	Vaginal, urine/urethral, cervical/endocervical, and rectal (861)	NAAT (NA)	82 (9.5%)
Munson et al., 2012 [67]	USA	Retrospective	F	Vaginal, urine/urethral, and cervical/endocervical (7277)	TMA (16S rRNA)	54.2%
Mishra et al., 2025 [68]	India	Prospective observational	F	Vaginal (114)	PCR (TVK3/TVK7)	5 (4.4%)
Madico et al., 1998 [69]	USA	NA	F	Vaginal (23)	PCR (β‐tubulin)	22 (96%)
Mayta et al., 2000 [70]	Peru	NA	F	Vaginal and urine/urethral (733)	PCR (18S rRNA)	56
Munson et al., 2013 [71]	USA	Retrospective	M	Urine/urethral (622)	TMA (16S rRNA)	6.6%
Mao et al., 2015 [72]	China	NA	F	Vaginal and urine/urethral (32)	PCR (18S rRNA)	27
Matini et al., 2017 [73]	Iran	NA	F	Vaginal (862)	RFLP (Tv8S/Tv9R and Tv10S/Tv11R)	16 (1.9%)
Matini et al., 2012 [74]	Iran	NA	F	Vaginal (50)	PCR‐SSCP (5.8S rRNA)	42 (84%) and 8 (16%)
Matini et al., 2014 [75]	Iran	NA	F	Vaginal (50)	PCR‐SSCP (actin‐S1/actin‐As1 and actin‐S2/actin‐As2)	425 and 492 bp nucleotide fragments of actin gene
Mitchev et al., 2021 [76]	South African	NA	F	Vaginal (250)	PCR (NA)	19
Masha et al., 2017 [77]	Kenya	Cross‐sectional	F	Vaginal (349)	RFLP (Tv8S/Tv9R)	43.5%
Morris et al., 2021 [78]	USA	Cross‐sectional	F	Vaginal (1585)	PCR (18S rRNA)	1449 (93.2%)
Margarita et al., 2019 [79]	Italy	NA	F	Vaginal (48)	Real‐time PCR (NM)	51
Momeni et al., 2015 [80]	Iran	NA	F	Vaginal (45)	PCR‐RFLP (Tv8S/Tv9R and Tv10S/Tv11R)	8 genotypes
Noh et al., 2019 [81]	Korea	NA	F	Urine/Urethral (4)	PCR (TV‐E650‐1)	4 and 1
Nye et al., 2009 [82]	USA	Prospective	F and M	Vaginal, urine/urethral, and cervical/endocervical (594)	TMA and PCR (β‐tubulin)	In women = PCR (73) and ATV (87); in men = PCR (24) and ATV (41)
Nabweyambo et al., 2017 [83]	Uganda	Cross‐sectional	F	Vaginal (150)	PCR (AP65)	12 (8%)
Orujzadeh et al., 2019 [84]	Iran	NA	F	Vaginal (1700)	PCR‐RFLP (Tv8S/Tv9R and Tv10S/Tv11R)	10 genotypes
Özel et al., 2023 [85]	Turkey	NA	F	Vaginal (200)	PCR (β‐tubulin (btub1))	8 (4%)
Pillay et al., 2007 [86]	South Africa	NA	F	Vaginal and urine/urethral (119)	Real‐time PCR, PCR (PCR (TVK3/TVK7), and real‐time PCR (β‐tubulin))	PCR (65.5% for vaginal swabs and 36.9% for urine) and real‐time PCR (68.9% for vaginal swabs and 61.3% for urine)
Price et al., 2018 [87]	South Africa	NA	F	Vaginal (359)	PCR (NA)	76 (20%)
Paul et al., 2012 [88]	India	Prospective	F	Vaginal (198)	PCR (TVA5/TVA6)	10 (5.02%)
Patil et al., 2012 [89]	Belgium	Cross‐sectional	F	Vaginal (200)	PCR (TVK3/TVK7)	60 (30%)
Queza et al., 2013 [90]	Philippines	NA	F	Vaginal and urine/urethral (216)	PCR (TFR1/2, TVK3/7, and TV1/2)	15
Radonjic et al., 2006 [91]	Serbia and Montenegro	NA	F	Vaginal (200)	PCR (BTUB 9/BTUB 2)	22 (11.0%)
Riley et al., 1992 [92]	USA	NA	F and M	Vaginal and urine/urethral (24)	PCR (TVA5/TVA6)	12
Ryu et al., 1999 [93]	Korea	NA	F	Vaginal (426)	PCR (TV‐E650)	23
Roy et al., 2023 [94]	Bangladesh	Cross‐sectional descriptive	F	Cervical/endocervical (102)	PCR (designed primer set)	6 (5.9%)
Rabie et al., 2012 [95]	Iran	Cross‐sectional	F	Vaginal (683)	PCR (TVK3/TVK7 and TVA5‐1/TVA6)	15 (2.2%)
Rumyantseva et al., 2015 [96]	Sweden	NA	F and M	Vaginal and urine/urethral (1261)	Real‐time PCR (Tv‐E650)	1
Rivera et al., 2009 [97]	Philippines	NA	F	Vaginal (57)	PCR (5.8S rDNA and ITS1/ITS2)	Six sequence types (H1 = 46, H4 = 7, H2, H3, H5, and H6 = 1)
Rivera et al., 2017 [98]	Philippines	NA	F	Vaginal (772)	RT‐PCR (TVV1F2875‐TVV1R3443, TVV2F2461‐TVV2, TVV3F61‐TVV3R482, and TVV4F1338‐TVV4R1834)	96
Salazar et al., 2019 [99]	Spain	NA	F and M	Vaginal, urine/urethral, and cervical/endocervical (622)	Real‐time PCR (NM)	10
Saleh et al., 2014 [100]	Sudan	Cross‐sectional	F	Vaginal (297)	PCR (TV3/TV7)	253 (85.2%)
Schirm et al., 2007 [101]	Netherlands	NA	F and M	Vaginal and urine/urethral (2071)	Real‐time PCR (β‐tubulin)	40
Seo et al., 2014 [102]	Korea	NA	M	Urine/urethral (201)	PCR (TVK3/TVK7 and BTUB 9/BTUB 2)	8 (4%)
Schwebke et al., 2011 [103]	USA	Prospective	F	Vaginal, urine/urethral, and liquid cytology/ThinPrep, and cervical/endocervical (933)	TMA (18s rRNA)	Vaginal swab = 12.7%, urine = 11.4%, endocervical swab = 12.4%, and ThinPrep = 11.4%
Smith et al., 2005 [104]	Australia	NA	F	Vaginal, urine/urethral, and cervical/endocervical (205)	PCR (β‐globin)	24%
Sutton et al., 2007 [105]	USA	NA	F	Vaginal (3754)	PCR (NA)	3.1%
Shaio et al., 1997 [106]	Taiwan	NA	F	Vaginal (96)	Nested PCR (OP1/OP2 and IP1/IP2)	14
Surya et al., 2024 [107]	India	Prospective observational	F	Vaginal (600)	PCR (TVK3/TVK7, BTUB 9/BTUB 2, and AP65)	9 (1.5%)
Simpson et al., 2007 [108]	Australia	NA	F	Vaginal (500)	Real‐time PCR (β‐tubulin and 18S rRNA)	29
Souza et al., 2013 [109]	Brazil	NA	F	Vaginal andcervical/endocervical (556)	Multiplex PCR (β‐globin)	7 STIs
Sutcliffe et al., 2010 [110]	USA	NA	F	Vaginal and urine/urethral (507)	BTUB real‐time PCR (β‐tubulin)	48
Tayoun et al., 2015 [111]	USA	NA	F	Vaginal, urine/urethral, and rectal (83)	Multiplex PCR (2‐kb)	8
Tipple et al., 2016 [112]	UK	Prospective	F	Vaginal (901)	BDQx (NM)	11 (1.2%)
Testardini et al., 2016 [113]	Argentina	NA	F	Vaginal (386)	PCR (18S rRNA and β‐globin)	24 (6.2%)
Tavakoli Oliaee et al., 2017 [114]	Iran	Descriptive cross‐sectional	F	Vaginal and urine/urethral (150)	RFLP (Tv8S/Tv9R and Tv10S/Tv11R)	24
Vahidnia et al., 2014 [115]	Netherlands	NA	F and M	Vaginal and urine/urethral (787)	FLOW and Viper (repeated DNA target)	15
Van Der Pol et al., 2014 [116]	USA	NA	F	Vaginal (838)	TVQ and ATV (ap65‐1)	116
Van Der Pol et al., 2006 [117]	USA	NA	F and M	Vaginal, urine/urethral, and cervical/endocervical (677)	PCR (TVA5/TVA6, TVK3/TVK7, and β‐Tub2/β‐Tub9)	97
Van Der Schee et al., 1999 [118]	Netherlands	Prospective	F and M	Vaginal and urine/urethral (470)	PCR (TVK3/TVK7)	82
Van der Veer et al., 2016 [119]	Netherlands	Cross‐sectional	F	Vaginal, urine/urethral, and cervical/endocervical (87)	MLST and nested PCR (*Tryp, glut, ft2a, alts, dmrp, shmt,* and *m6pi*)	71 (81.6%)
Valadkhani et al., 2010 [120]	Iran	NA	F	Vaginal, & Urine/Urethral (161)	PCR (β‐tubulin)	7 (4.3%)
Vatanshenassan et al., 2010 [121]	Iran	NA	F	Vaginal and urine/urethral (500)	PCR (CP4)	12 (2.4%)
Waaij et al., 2016 [122]	South Africa	Cross‐sectional	F	Vaginal and rectal (615)	Real‐time PCR (NA)	207
Wendel et al., 2002 [123]	USA	Cross‐sectional	F and M	Vaginal, urine/urethral, and cervical/endocervical (28)	PCR (dsRNA)	21 (75%)
Wendel et al., 2002 [124]	USA	Observational	F	Vaginal (337)	PCR (β‐tubulin)	97
Yarizadeh et al., 2021 [125]	Iran	Cross‐sectional	M	Urine/urethral (214)	PCR (TVK3/TVK7)	10 (4.7%)
Yar et al., 2017 [126]	Turkey	NA	M	Urine/urethral (138)	Nested PCR (TVC3F/TVC4R and TVC11F/TVC12R)	9 (6.5%)
Ziaei Hezarjaribi et al., 2020 [127]	Iran	NA	F and M	Vaginal and urine/urethral (3500)	Nested PCR (Ops and IPs)	17 (0.48%)
Zandijk et al., 2009 [128]	Netherlands	NA	NA	Urine/urethral (963)	Real‐ time PCR (β‐tubulin)	8 (0.3%)
Zhang et al., 2023 [129]	China	NA	M	Urine/urethral and semen/prostate fluid (634)	Nested PCR (Tv8S, Tv9R, Tv10S, and Tv11R)	32 (5.05%)
Zhang et al., 2018 [130]	China	NA	F	Vaginal (267)	PCR‐RFLP (Ops and IPs)	68

Abbreviations: ATV = APTIMA Trichomonas vaginalis, BDQx = Becton Dickinson Qx, F = female, FLOW = Roche Aurora FLOW, LAMP = loop‐mediated isothermal amplification, M = male, MLST = multilocus sequence typing, NA = information not available in the published article, NAAT = nucleic acid amplification test, PCR = polymerase chain reaction, PCR‐SSCP = PCR‐single‐stranded conformational polymorphism, qPCR = quantitative real‐time polymerase chain reaction, RAPD = random amplified polymorphic DNA, RFLP = restriction fragment length polymorphism, RT‐PCR = reverse transcription polymerase chain reaction, TMA = Gen‐Probe’s transcription‐mediated amplification, TV = trichomonas vaginalis, TVQ = T. vaginalis Qx, Viper = nucleic automated system.

Studies were conducted across nearly all continents. The highest number originated from Asia (43 studies), followed by North America (36 studies), Europe (18 studies), Africa (14 studies), South America (6 studies), and Oceania (4 studies).

At the country level, the United States (*n* = 33) and Iran (*n* = 16) contributed the largest number of studies, followed by South Korea (*n* = 8), India (*n* = 6), and China, the Netherlands, and South Africa (*n* = 5 each). The Philippines and Australia each contributed 4 studies, while the United Kingdom contributed 3 studies. Taiwan, Brazil, Cuba, Egypt, Turkey, Spain, and Italy each contributed 2 studies. The remaining countries, including Ivory Coast, Congo, Zambia, Sri Lanka, Chile, Georgia, Mexico, Peru, Kenya, Uganda, Serbia and Montenegro, Bangladesh, Sweden, Sudan, and Argentina, each contributed 1 study.

In total, 80,476 samples were analyzed across the included studies. Vaginal samples were the most frequently used (61 studies). Twenty‐three studies used both vaginal and urine/urethral samples, while 14 used urine/urethral samples alone. Eight studies used vaginal, urine/urethral, and cervical/endocervical samples. Cervical/endocervical samples alone were used in 2 studies. Various combinations were also reported, including urine/urethral and cervical/endocervical (2 studies), vaginal and cervical/endocervical (2 studies), vaginal and semen/prostatic fluid (1 study), vaginal, urine/urethral, cervical/endocervical, and rectal (1 study), vaginal, urine/urethral, and rectal (1 study), vaginal and rectal (1 study), and urine/urethral and semen/prostatic fluid (1 study). Liquid‐based cytology (ThinPrep) samples were used in 1 study, either alone or in combination.

Of the included studies, 91 focused only on women, 7 on men, 19 on both genders, and 2 did not specify the participants’ gender.

PCR was the most frequently used molecular diagnostic method (58 studies), followed by real‐time PCR (12 studies). Nested PCR and TMA were each reported in 5 studies, while PCR‐RFLP was used in 4 studies. Affirm VPIII, multiplex PCR, and RFLP were each applied in 3 studies. RAPD, RT‐PCR, NAAT, and PCR‐SSCP were each reported in 2 studies.

Several studies employed combinations of diagnostic methods, most commonly PCR with real‐time PCR (2 studies). Other combinations included PCR with LAMP, PCR with nested PCR, TMA with PCR, and various platform‐based assays such as APTIMA TV, Xpert TV, BTUB real‐time PCR, BDQx, FLOW, Viper, TVQ, ATV, and MLST (each reported in one study).

Among studies reporting performance metrics, sensitivity ranged from 46.3% to 100%, with 18 studies reporting 100% sensitivity. Specificity was consistently high, with 20 studies reporting 100% specificity (Table 3). PCR, real‐time PCR, ATV assay, LAMP, Xpert TV, TMA, multiplex PCR, and BTUB PCR were most frequently associated with perfect or near‐perfect performance metrics.

**TABLE 3:** The sensitivity and specificity of molecular methods were investigated in the studies.

Authors/years/references	Molecular methods	Sample sizes	Sensitivity (%)	Specificity (%)
Andrea et al., 2011 [15]	Affirm VPIII assay	766	Affirm VPIII assay = 63.4	Affirm VPIII assay = 99.9
	ATV assay		ATV assay = 100	ATV assay = 100

Bui et al., 2023 [18]	Real‐time PCR	136	100	100

Caliendo et al., 2005 [22]	Real‐time PCR	524	100	99.6

Cartwright et al., 2013 [23]	Affirm VPIII assay	320	46.3	100

Crucitti et al., 2003 [28]	PCR	416	92.8	100

Crucitti et al., 2008 [29]	PCR	160	94.4	NA

DeMeo et al., 1996 [30]	Affirm VPIII assay	615	89.5	99.8

Gaydos et al., 2016 [37]	APTIMA TV	990	90.7	98.9

Goo et al., 2016 [38]	Multiplex PCR	50	Multiplex PCR = 57.5	Multiplex PCR = 100
	LAMP		LAMP = 100	LAMP = 70

Hardick et al., 2006 [39]	BTUB PCR	611	BTUB PCR = NA	BTUB PCR = NA
	TMA		TMA = 96.7	TMA = 97.5

Hardick et al., 2003 [40]	BTUB PCR	253	90.1	100

Huppert et al., 2007 [43]	TMA	330	98.2	98

Herath et al., 2021 [45]	PCR	385	TFR1/2 = 100	100
			TV16Sf/r = 88.20 TVK3/7 = 76.50	

Jones et al., 2013 [50]	PCR	925	76.7	99.1

Jordan et al., 2001 [51]	PCR	552	97.8	97.4

Kaydos et al., 2002 [54]	PCR	1513	66.9	98.3

Kaydos‐Daniels et al., 2003 [55]	PCR	1225	92.7	88.6

Khurana et al., 2019 [56]	PCR	153	PCR = 100	PCR = 100
	LAMP		LAMP = 100	LAMP = 100
	Xpert TV		Xpert TV = 100	Xpert TV = 99.18

Lawing et al., 2000 [61]	PCR	190	89	97

Mishra et al., 2025 [68]	PCR	114	100	98.2

Madico et al., 1998 [69]	PCR	23	BTUB 9/2 = 97	BTUB 9/2 = 97.8
			TVA 5‐1/2 = 69.7	TVA 5‐1/2 = 100

Mayta et al., 2000 [70]	PCR	733	Vaginal swab = 100	Vaginal swab = 98 and urine = 99.7
			Urine = 100	

Mitchev et al., 2021 [76]	PCR	250	100	99.59

Nabweyambo et al., 2017 [83]	PCR	150	91.7	99.3

Pillay et al., 2007 [86]	PCR	119	PCR (vaginal = 100, urine = 56.4)	PCR (vaginal = 100, urine = 97.6)
	Real‐time PCR		Real‐time PCR (vaginal = 100, urine = 76.7)	Real‐time PCR (vaginal = 82.9, urine = 97.0)

Paul et al., 2012 [88]	PCR	198	100	98

Queza et al., 2013 [90]	PCR	216	TFR1/2 = 78.8	TFR1/2 = 100
			TVK3/7 = 97	TVK3/7 = 100
			TV1/2 = 66.7	TV1/2 = 100

Radonjic et al., 2006 [91]	PCR	200	80.95	97.21

Ryu et al., 1999 [93]	PCR	426	100	100

Rumyantseva et al., 2015 [96]	Real‐time PCR	1261	100	100

Salazar et al., 2019 [99]	Real‐time PCR	622	100	100

Schirm et al., 2007 [101]	Real‐time PCR	2071	100	99.9

Schwebke et al., 2011 [103]	TMA	933	Vaginal swabs = 100	Vaginal swabs = 99
			Endocervical swabs = 100	Endocervical swabs = 99.4
			ThinPrep = 100	ThinPrep = 99.6
			Urine = 95.2	Urine = 98.9

Smith et al., 2005 [104]	PCR	205	PCR (HVS = 96, LVS = 94, urine = 74)	PCR (HVS = 99.4, LVS = 99.4, urine = 99.3)
	TMA			

Surya et al., 2024 [107]	PCR	600	TVK 3/7 = 100	TVK 3/7 = 100
			AP65 = 66.67	AP65 = 100
			BTUB 9/2 = 66.67	BTUB 9/2 = 100

Souza et al., 2013 [109]	Multiplex PCR	556	100	100

Testardini et al., 2016 [113]	PCR	386	83.3	100

Van Der Pol et al., 2014 [116]	TVQ	838	98.3	99

Van Der Pol et al., 2006 [117]	PCR	677	Vaginal swabs (TVA = 48.7, TVK = 79.5)	Vaginal swabs (TVA = 100, TVK = 97.8)
			Urine (TVK = 96, β‐Tub = 92)	Urine (TVK = 99.2, β‐Tub = 99.4)

Vatanshenassan et al., 2010 [121]	PCR	500	80	99.6

Waaij et al., 2016 [122]	Real‐time PCR	615	Presto^plus^ = 95.28	Presto^plus^ = 100
			LightMix Kit = 98.1	LightMix Kit = 98.4

Wendel et al., 2002 [124]	PCR	28	84	94

Abbreviations: ATV = APTIMA Trichomonas vaginalis, BDQx = Becton Dickinson Qx, FLOW = Roche Aurora FLOW, HVS = high vaginal swab, LAMP = loop‐mediated isothermal amplification, LVS = low vaginal swab, MLST = multilocus sequence typing, NA = information not available in the published article, NAAT = nucleic acid amplification test, PCR = polymerase chain reaction, PCR‐SSCP = PCR single‐stranded conformational polymorphism, qPCR = quantitative real‐time polymerase chain reaction, RAPD = random amplified polymorphic DNA, RFLP = restriction fragment length polymorphism, RT‐PCR = reverse transcription polymerase chain reaction, TMA = Gen‐Probe’s transcription‐mediated amplification, TV = trichomonas vaginalis, TVQ = T. vaginalis Qx, Viper = nucleic automated system.

## 4. Discussion

In the past decade, researchers have increasingly prioritized diagnostic methods that enhance the detection of pathogenic microorganisms. *T. vaginalis* is typically diagnosed using vaginal discharge or urine samples analyzed by wet‐mount microscopy, culture, or molecular techniques, each of which has distinct advantages and limitations. While culture methods are often time‐consuming, they are still considered the gold standard for *T. vaginalis* identification. Nonetheless, wet‐mount microscopy is the most commonly used diagnostic test due to its low cost, despite its limited sensitivity. Molecular techniques offer greater sensitivity than culture and wet‐mount microscopy for laboratory diagnosis of *T. vaginalis*. However, their broad use for routine detection of *T. vaginalis* in resource‐limited settings is restricted by financial constraints. Babafemi et al. have reported that the sensitivity of culture methods compared to molecular diagnostics ranges from 34.9% to 78%, while their specificity is typically 100%. Also, wet‐mount microscopy demonstrated higher specificity but lower sensitivity, ranging from 34.2% to 58.5% [9]. Patel et al. have recommended using culture methods when wet‐mount microscopy results are negative. They emphasized that the sensitivity of culture methods should be interpreted with caution, because of variations among different culture media that can influence diagnostic outcomes [131].

This systematic review examined 119 studies, with PCR and real‐time PCR identified as the most frequently employed molecular methods reported in 58 and 12 studies, respectively. The literature review highlighted PCR and related nucleic acid amplification techniques were among the most accurate and widely used molecular methods for diagnosing *T. vaginalis* and are frequently considered reference molecular assays in diagnostic studies. Compared to traditional methods such as cultivation, immunologic assays, and microscopy, molecular diagnostics offer superior sensitivity and specificity. Babafemi et al. (2025) have emphasized that molecular techniques are sensitive, specific, rapid, and reproducible [9]. Rahmani et al. (2021) have noted that recent advances in rapid molecular tests, such as PCR, GeneXpert, AmpliVue, OSOM, and the APTIMA TV assay, have significantly improved diagnostic sensitivity and specificity, making them ideal tools for clinical use [132]. PCR utilizes sequence‐specific enzymes to amplify and detect targeted regions of *T. vaginalis* DNA and is considered the most precise method for laboratory‐based diagnosis. The GeneXpert *T. vaginalis* assay is a highly accurate and sensitive diagnostic method capable of detecting *T. vaginalis* in both urine specimens and vaginal swabs from symptomatic and asymptomatic women. The AmpliVue assay employs helicase‐dependent amplification (HDA) technology and identifies *T. vaginalis* through a three‐step procedure completed within 45 min in both symptomatic and asymptomatic individuals. The OSOM Trichomonas rapid test is a highly sensitive and accurate point‐of‐care assay that detects *T. vaginalis* in five steps within approximately 10 min. The APTIMA *T. vaginalis* assay targets specific ribosomal RNA (rRNA) sequences and applies transcription‐mediated amplification (TMA), followed by detection of amplified products using specialized high‐complexity instrumentation operated by trained laboratory personnel. Consequently, APTIMA is not suitable for point‐of‐care testing and is more costly than many non‐nucleic acid amplification test (NAAT)‐based methods. Overall, among the available diagnostic approaches, the OSOM Trichomonas test, GeneXpert TV assay, AmpliVue assay, and APTIMA TV assay are considered optimal due to their high accuracy and sensitivity (13). Regarding time to result, OSOM provides results in approximately 10 min, whereas AmpliVue requires around 45 min. In terms of regulatory status, OSOM and GeneXpert TV assays are FDA‐cleared and/or CE‐marked depending on the region, while AmpliVue is also commercially approved for diagnostic use in several regulatory jurisdictions. With respect to suitability for low‐resource settings, OSOM is particularly advantageous as a point‐of‐care test requiring minimal equipment, whereas GeneXpert and AmpliVue require specialized instruments and are therefore more suitable for well‐equipped laboratory settings with higher operational costs. Edwards et al. recommended NAATs in asymptomatic patients with low microbial loads, which may not be detectable by less sensitive methods [133]. PCR remains the workhorse method for diagnosing *T. vaginalis* for the following reasons: its proven high sensitivity and specificity across diverse sample types, widespread availability in diagnostic laboratories, long‐standing experience and familiarity among clinical personnel, cost‐effectiveness relative to some newer NAATs, and adaptability to various clinical contexts. Despite their advantages, PCR‐based diagnostics have limitations, including the inability to differentiate between live and dead organisms in previously treated patients, as well as limited accessibility in many diagnostic centers [131]. In this review, molecular‐based tests demonstrated high diagnostic performance, with 18 studies reporting 100% sensitivity and 20 studies reporting 100% specificity, and PCR, real‐time PCR, ATV assay, LAMP, Xpert TV, TMA, multiplex PCR, and BTUB PCR were most frequently associated with perfect or near‐perfect performance metrics. These findings suggest that molecular diagnostics are fast, accurate, and reliable and can serve as strong alternatives or complements to conventional methods such as culture or wet‐mount microscopy particularly in settings equipped with advanced technology. Nonetheless, some studies reported sensitivity and specificity below 100%, possibly due to low parasite loads or sampling errors, DNA extraction methods, primer/target gene differences, insufficient expertise in the use of molecular tests, and lack of funding, especially in third world countries.

The most commonly used sample types were vaginal swabs, reported in 61 studies, and urine samples, reported in 18 studies, which are accessible and practical for diagnostic purposes. Vaginal swabs showed high sensitivity and specificity when used with molecular techniques, making them cost‐effective and reliable in clinical and research settings. Urine samples also provided rapid results and were well‐suited for molecular testing. Notably, most current diagnostic protocols depend on urine and vaginal samples collected from women, while male‐specific diagnostic methods, such as urethral swabs, semen, or prostatic fluid, remain underdeveloped and less frequently utilized. This disparity can be attributed to the often‐asymptomatic nature of *T. vaginalis* infection in men, logistical and ethical challenges in obtaining prostatic or semen samples, and historical research emphasis on women’s reproductive health. Addressing this gap through development and validation of male‐specific diagnostic protocols are essential to achieve a more comprehensive understanding of *T. vaginalis* epidemiology across sexes [132].

Of the 119 studies, 91 focused on women, 7 on men, and 19 on both genders. This trend shows the affinity of the parasite for the female reproductive tract, which provides a warm, moist environment conducive to infection. In addition, as *T. vaginalis* is mainly transmitted through sexual contact, women are more likely to exhibit symptoms, facilitating diagnosis and treatment. *T. vaginalis* infection in women is more frequently symptomatic and is associated with clinically significant reproductive and obstetric outcomes, prompting targeted screening and research efforts. In contrast, *T. vaginalis* infection in men is often asymptomatic or self‐limiting, leading to underdiagnosis and limited routine testing. Additionally, the historical prioritization of women’s reproductive health, particularly in the context of STIs, has contributed to a research bias favoring female‐only study design. Collectively, these factors help explain the marked gender disparity observed in this review and highlight an important gap in current knowledge regarding the epidemiology and clinical significance of *T. vaginalis* infection in men [134]. Among the 119 studies reviewed, the United States (*n* = 33), Iran (*n* = 16), and South Korea (*n* = 8) contributed the highest number of studies, thereby reflecting a clear geographic imbalance in research output. In contrast, relatively few studies originated from regions such as Oceania and South America, which may be attributed to limited access to sensitive diagnostic methods, underreporting of nonviral STIs, lower prioritization of *T. vaginalis* within public health agendas, and constraints in research funding. While a greater number of studies does not necessarily correlate with higher infection rates, *T. vaginalis* remains among the most prevalent STIs in the United States. In this country, factors contributing to elevated infection rates among certain populations entail high‐risk sexual behaviors, socioeconomic disparities, and racial inequalities [135]. Conversely, the lack of studies from high‐prevalence regions limits the external validity and global generalizability of diagnostic performance data, as most molecular assays have been validated primarily in resource‐rich settings. This underrepresentation poses a significant challenge for global diagnostic standardization, as test performance, feasibility, and implementation may differ in settings with limited resources, emphasizing the need for expanded epidemiological and diagnostic research in underrepresented, high‐burden regions. For example, Africa is recognized as a high‐prevalence region for *T. vaginalis* infection, yet only 14 studies originated from this region, underscoring a substantial imbalance between disease burden and research output.

Several strengths of this systematic review are its comprehensive search strategy across multiple databases and the inclusion of a large number of studies. Additionally, the two independent reviewers extracted data with a 97% consensus on the papers they selected separately, using the JBI Critical Appraisal Checklist to ensure study quality, and this is the first systematic review related to the use of all molecular techniques for the detection of *T. vaginalis*, which can provide baseline data for policy makers and researchers on health policy planning. However, limitations include restriction to English‐language publications and differences in the performance of molecular tests due to variations in diagnostic protocols and target genes, often influenced by the choice of commercial diagnostic kits, factors that may affect the generalizability of findings. One of the most important limitations of this study relates to the overall reporting quality of the included literature. Specifically, methodological details were unclear in 83 studies. This issue was primarily attributable to the inadequate or insufficiently detailed reporting of methodological sections in the original articles, rather than limitations in our data extraction process. Therefore, this finding reflects a broader and well‐recognized challenge of suboptimal reporting standards within the field. We have accordingly highlighted this as a key limitation of the available evidence base. Also, the review was narrative and did not perform a meta‐analysis (which could be a limitation due to heterogeneity across studies, including differences in study design, populations, diagnostic protocols, and outcome measures).

## 5. Conclusion

Molecular tests, despite certain limitations, are increasingly recommended as alternatives or complementary tools to traditional diagnostic methods especially in clinical settings due to their ability to detect *T. vaginalis* more rapidly and accurately. Accumulating evidence demonstrates that molecular tests consistently outperform microscopy, culture, and immunoassays, particularly in asymptomatic infections and cases with low parasite burden. Consequently, where laboratory infrastructure and financial resources permit, molecular methods should be prioritized as the preferred diagnostic approach for *T. vaginalis* to improve case detection, surveillance accuracy, and treatment outcomes. However, until these tools become simpler and more affordable, diagnostic results should be interpreted with caution, and future investigations should address this challenge. Therefore, further studies are recommended in male populations with standardized sampling protocols, in high‐prevalence and resource‐limited settings, as well as comparative studies of newer, simpler NAATs/LAMP versus conventional PCR. Additionally, evaluations of cost‐effectiveness in different contexts are warranted. It should be emphasized that a coordinated call to action is also necessary, including the standardization of diagnostic protocols, the integration of molecular testing into national STI surveillance programs, and investment in diagnostic capacity in low‐resource settings.

## Author Contributions

All authors have contributed to the result interpretation and manuscript drafting and approved the submission of the final copy for publication.

## Funding

This article was financially supported by the Ilam University of Medical Sciences, Ilam, Iran.

## Ethics Statement

The protocol was approved by the Ethics Committee of Ilam University of Medical Sciences (IR. MED Ilam.REC.1403.120).

## Conflicts of Interest

The authors declare no conflicts of interest.

## Data Availability

The data that support the findings of this study are available on request from the corresponding author. The data are not publicly available due to privacy or ethical restrictions.
